# Lysosomal dysfunction in diabetic cardiomyopathy

**DOI:** 10.3389/fragi.2023.1113200

**Published:** 2023-01-20

**Authors:** Satoru Kobayashi, Younghee Hahn, Brett Silverstein, Mandeep Singh, Adeline Fleitz, Jennifer Van, Hongling Chen, Qiangrong Liang

**Affiliations:** Department of Biomedical Sciences, College of Osteopathic Medicine, New York Institute of Technology, New York, NY, United States

**Keywords:** diabetes, cardiovascular, lysosome, autophagy, lysosomal membrane damage, cardiomyopathy

## Abstract

Diabetes is a major risk factor for a variety of cardiovascular complications, while diabetic cardiomyopathy, a disease specific to the myocardium independent of vascular lesions, is an important causative factor for increased risk of heart failure and mortality in diabetic populations. Lysosomes have long been recognized as intracellular trash bags and recycling facilities. However, recent studies have revealed that lysosomes are sophisticated signaling hubs that play remarkably diverse roles in adapting cell metabolism to an ever-changing environment. Despite advances in our understanding of the physiological roles of lysosomes, the events leading to lysosomal dysfunction and how they relate to the overall pathophysiology of the diabetic heart remain unclear and are under intense investigation. In this review, we summarize recent advances regarding lysosomal injury and its roles in diabetic cardiomyopathy.

## 1 Introduction

Diabetes is a major risk factor for the development of various cardiovascular complications, which constitute the leading causes of mortality in both type 1 and type 2 diabetic populations. Moreover, diabetic patients have an especially poor prognosis following myocardial infarction (Paulson, 1997; Haffner et al., 1998; Heather et al., 2022). In addition to increased prevalence of atherosclerosis and hypertension, a heart muscle-specific disease that is independent of vascular pathology, known as diabetic cardiomyopathy, has been recognized as an important risk factor for heart failure and mortality in diabetic patients (Bell, 1995; Boudina and Abel, 2007; Ritchie and Abel, 2020). Overall, diabetic hearts display abnormal metabolism, progressive deterioration of contractile function, and varying degrees of hypertrophy and fibrosis.

Oxidative stress has been thought to be the major mechanism that mediates diabetic cardiomyopathy (Wold et al., 2005; Packer, 2020), which is strongly supported by the ability of various antioxidants to reduce diabetic heart injury in animal studies (Liang et al., 2002; Ye et al., 2003; Fiordaliso et al., 2004; Ye et al., 2004; Wold et al., 2005). However, clinical trials supplementing antioxidants have failed to provide a positive outcome in the management of cardiovascular diseases (Lonn et al., 2002; Sacco et al., 2003; Lonn et al., 2005; Sesso et al., 2012; Schwingshackl et al., 2017; Pickering et al., 2018). This discrepancy suggests that a more thorough understanding of the cellular and molecular mechanisms underlying diabetic cardiomyopathy and heart failure is needed.

Since cardiac myocytes can hardly be regenerated by proliferation, there must exist intracellular mechanisms that not only deal with the stressors *per se*, but also repair or remove injured intracellular contents resulting from the stressors (Terman et al., 2008). The Autophagy-Lysosome system plays a central role in eliminating and recycling cellular materials to maintain cellular homeostasis (Pivtoraiko et al., 2009; Ornatowski et al., 2020). The lysosome is the site responsible for the degradation of intracellular debris that may pose a risk of cytotoxicity. It is becoming clear that lysosomes themselves can be the target of stressors, such as oxidative stress, and can act as signaling hubs that induce cell death. In the diabetic heart, autophagic activity and lysosomal structure and degradative enzyme activity are all altered (Mellor et al., 2011a; Mellor et al., 2011; Xie et al., 2011; Xu et al., 2013). However, the relationship between autophagy dysfunction and lysosomal alteration as well as their roles in diabetic cardiac injury remain partially understood. The goal of this review is to summarize recent research findings regarding diabetes-induced lysosomal dysfunction and to explore the possibility of targeting lysosomes for the treatment of diabetic cardiomyopathy.

## 2 The structure, function, and regulation of lysosomes in the heart

**FIGURE 1:**
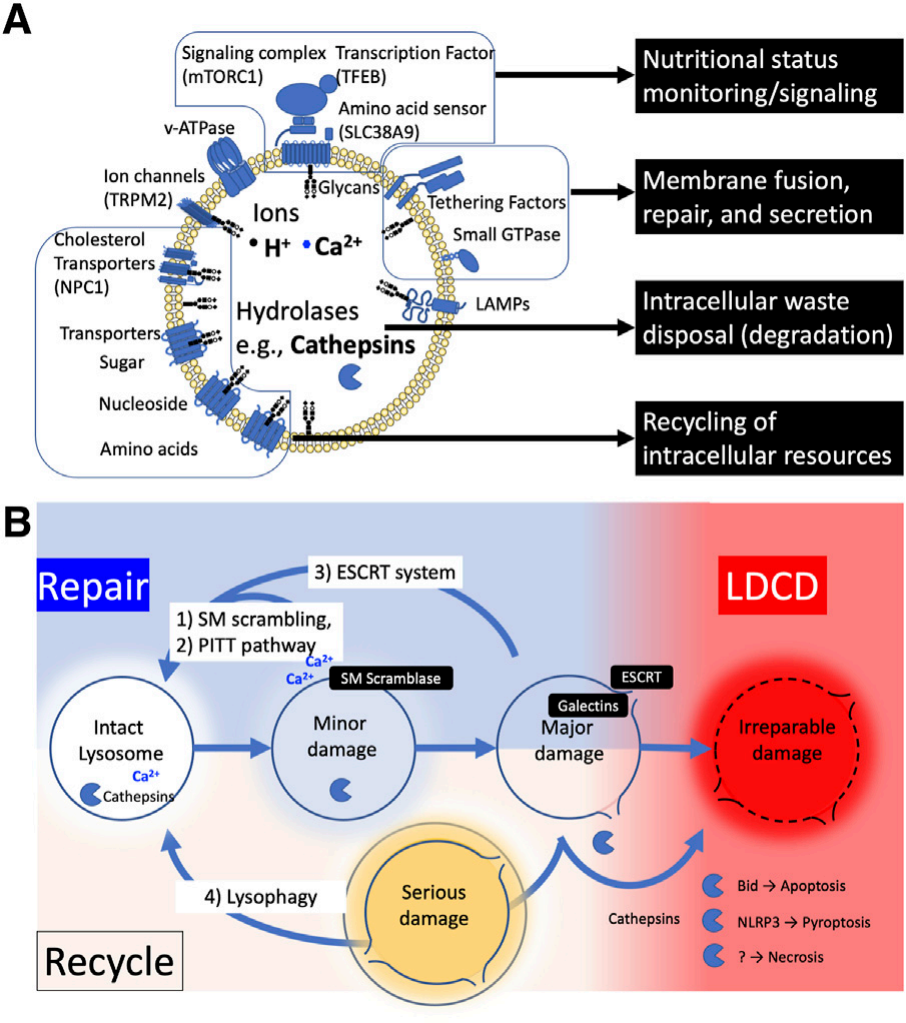
**(A)** The structure and functions of lysosome. **(B)** The fate of injured lysosome. Damaged lysosomes can be repaired or recycled; 1) Mild damage can trigger the sphingomyelin (SM) scrambling to reseal the ruptured lipid membrane by reversing the balanced sphingomyelin distribution of membrane lipids inside and outside (SM scrambling). 2) Or the injured membrane is tagged with phosphatidylinositol-4-phosphate (PtdIns4P), which recruits lipid-binding protein ORPs to form a micro contact domain between ER and lysosome membrane, promoting lipid transport to replace the membrane lipids (PITT). 3) Further damage allowing galectins to enter the lumen recruits ESCRT protein complexes to seal the membrane pores of the lysosome (ESCRT). 4) When lysosomes become seriously damaged and lysosomal enzymes are released into the cytoplasm, they are tagged and removed by lysophagy, and the digested components are recycled. When the cell fails to rescue the injured lysosomes, cathepsins are released into cytosol, resulting in various types of cell death.

The lysosome is a membrane-bound organelle that functions as the primary site of cellular degradation and recycling, varying in size and number depending on cell type and its environment (Figure 1A). (Ballabio and Bonifacino, 2020; de Araujo et al., 2020) It contains over 60 hydrolytic enzymes, which are capable of digesting intra-/extra-cellular biomolecules, including cellular debris, in the lumen. The lysosomal lumen is strictly separated from the cytoplasm and other organelles to prevent hydrolytic enzymes and sequestered intracellular debris from escaping the lysosome. Lysosomal proteins are heavily glycosylated with N- and O-linked glycans (Tokhtaeva et al., 2017) and are protected from degradation by proteases (Fukuda, 1991; Reggiori et al., 2021).

The substances that pass through the lysosomal membrane are controlled by a variety of lysosomal membrane proteins. For example, the interior of the organelle is maintained at a highly acidic pH (4.5–5.0), to maximize the efficiency of digestive enzymes. The acidic pH of the lysosomal lumen is maintained by multiple ion pumps and channels such as a proton pump Vacuolar H^+^-ATPase (v-ATPase), and a calcium channel TRPML1 embedded in the membrane (Zeng et al., 2020). In the process of autophagy, lysosomes fuse with the autophagosomes to provide degradative enzymes. In non-cardiomyocytes, syntaxin17 (STX17) is required for membrane fusion between autophagosome and lysosome, but STX17 is not required for autophagosome-lysosome fusion in human cardiomyocytes (Chi et al., 2019). Instead, the lysosomal membrane protein LAMP2B is essential for membrane fusion with autophagosome. In chaperone-mediated autophagy (CMA), specific proteins are selectively transported to lysosomes by the assistance of molecular chaperone Hsp73. The substrate-chaperone complex binds to lysosome-associated membrane protein 2a (LAMP2A) and enters the lysosome (Terman et al., 2008).

Lysosomes contribute to the uptake of nutrients and the supply and recycling of materials necessary for cellular homeostasis (Xu and Ren, 2015). To this end, lysosomes serve as signaling hub in response to environmental cues such as nutrients and stresses. The mechanistic target of rapamycin complex 1 (mTORC1) and transcription factor EB (TFEB) are the key mediators of these lysosomal adaptation. Although the detailed molecular mechanisms are not fully elucidated, it is believed that v-ATPase responds to the amino acid state of the lysosomal lumen and transmits information to mTORC1 *via* Ragulator. mTORC1 phosphorylates the serine 211 residues to promote TFEB degradation and inhibit nuclear translocation (Settembre and Ballabio, 2014; Rebsamen et al., 2015). Regulation of lysosomal function by acting on TFEB has major consequences for cell clearance and energy metabolism (Sardiello, 2016). Since cardiac lysosomes are important organelles that are not only required for intracellular waste removal, but also serve as nutrient and stress sensors, lysosomal quality and function must be strictly controlled and regulated.

## 3 Lysosomal injury and repairing mechanisms

To control the environment inside the organelle, the lysosome is tightly packed with sphingolipids (Tang et al., 2022). However, the lysosomal membrane can be damaged under stressful conditions. Lysosomal Membrane Permeabilization (LMP) is a condition in which substances pass through a permeable lysosomal membrane, allowing ions and enzymes that should be contained within the lysosomal lumen to leak into the cytosol. Conversely, LMP also refers to conditions where cytosolic elements that should be excluded from the lysosomal lumen translocate into the lysosome. A variety of cellular stresses can cause lysosomal damage leading to different degrees of lysosomal membrane permeabilization (LMP) (Boya and Kroemer, 2008; Terman et al., 2008; Pivtoraiko et al., 2009). The ways that LMP affects the ensuing lysosomal responses differ depending on the degree of stress applied to lysosomes. When the disruption of membrane integrity is relatively minor or caused by limited or moderate stress, lysosomal enzymes typically remain in the lumen. However, this can initiate ion leakage and pH changes within the lysosome, causing dysfunction of acidification and destabilization of lysosomal homeostasis (Ishida et al., 2013). Depending on the degree of the LMP, there are two types of responses that repair damaged lysosomes (Figure 1B): 1) Restoring limited membrane rupture, including endosomal sorting complex required for transport (ESCRT) machinery (Niekamp et al., 2022; Olmos, 2022), sphingomyelin scrambling (Niekamp et al., 2022), and phosphoinositide-initiated membrane tethering and lipid transport (PITT) pathway (Tan and Finkel, 2022); 2) Regenerating entire vesicles through autophagy (lysophagy and autophagy lysosome reformation ALR). (Nanayakkara et al., 2022).

It is becoming clear that there is a mechanism to repair minor injury in the lysosomal membrane that is not large enough to allow proteins to pass through. The endosomal sorting complex for transport (ESCRT) mechanism plays a key role in closing holes in the lysosomal membrane; the ESCRT complex forms spiral filaments at the fray of membrane that require suturing, to catalyze membrane remodeling, guided by ALG2-interacting protein X (ALIX)-ESCRT complex. A lysosomal membrane tension reduction and calcium ion leakage have been proposed as the cues triggering ESCRT assembly at the site of lysosomal injury. The ESCRT complex appears to be recruited to the site of injury upon detection of endo-lysosomal tension (Skowyra et al., 2018; Mercier et al., 2020). Calcium binding protein ALG2 and ALIX complex has been suggested to be responsible for recruiting the ESCRT complex (Skowyra et al., 2018), but other studies have not confirmed the need for ALIX (Lopez-Jimenez et al., 2018; Radulovic et al., 2018). The existence of rapid lysosomal membrane repair mechanisms has been also recently unveiled. One study has shown that upon LMP, sphingomyelin (SM) is exposed to cytosols, which induces rapid rearrangement of SM towards cytosol and lumen by the lysosome specific activity of Ca^2+^-dependent scramblases in ESCRT-independent manner (Niekamp et al., 2022). Another study showed that phosphatidylinositol-4 kinase type 2α (PI4K2A) accumulates in lysosomes with LMP and labels the injured lysosome with phosphatidylinositol-4-phosphate, thereby recruiting tethering proteins such as, oxysterol-binding protein (OSBP)-related protein (ORP) family, which triggers membrane displacement by contact with the ER membrane *via* phosphoinositide-initiated membrane tethering and lipid transport (PITT) pathway (Tan and Finkel, 2022). These lysosomal membrane repair mechanisms, including spontaneous repair of the lysosomal sphingolipids themselves, have just been discovered and further evidence is needed for such a response in cardiomyocytes.

When the damage to the membrane becomes more severe, to the extent that proteases and other macromolecules leak out, the glycan-binding protein galectins bind to the sugar chains (glucagon) in the lysosome and navigate ALIX complex to repair the membrane by the ESCRT system (Jia et al., 2020). At the same time, Galectin 8 interacts with sodium-coupled neutral amino acid transporter SLC38A and inactivates mTORC1, and Galectin 9 induces autophagy by binding to TAK and activating AMPK (Jia et al., 2019; Jia et al., 2020). Galecitin3 enters the lysosomal lumen, backs up the damaged membrane, and recruits TRIM16 (Kumar et al., 2017) and CUL4-RING ubiquitin ligases to induce lysophagy, which rapidly sequesters damaged lysosomes tagged with polyubiquitin into autophagosomes for degradation and regeneration (Lu et al., 2019). Autophagic lysosome reforming (ALR) is a mechanism that recycles lysosome fused to autophagosome (autolysosome) membranes to rederive lysosomes. In ALR, the autolysosome membrane is extruded along microtubules to generate membrane tubes called “reformation tubules.” The tubes are fragmented into “proto-lysosomes” and mature into functional lysosomes (Nanayakkara et al., 2022). As long as lysosomes can contain the degree of stress that damages themselves, the cellular responses strive to maintain cellular homeostasis. However, if the degree of damage is severe or accumulates beyond a threshold, cell death is induced as described later.

## 4 Lysosomal dysfunction and its impact on the heart

Due to the pleiotropic effects of the lysosome on cell death and survival, lysosomal dysfunction can cause various diseases through either decreased or excessive activity. Lysosomal storage diseases (LSDs) are often caused by the accumulation of substrates that have been incorporated into lysosomes and are not fully processed by the hydrolytic enzymes. Mutations or abnormalities in any one of the lysosomal enzymes can result in inadequate degradation or inability to process the accumulated substrates at the required pace. In Fabry disease and Krabbe disease, mutations in the degrading enzymes alpha-galactosidase (GLA) and galactocerebrosidase (GALC) both lead to cardiomyopathy (Platt et al., 2012). LSDs that cause cardiac problems are not limited to degradative enzyme abnormalities but can also be caused by defects in lysosomal protein targeting, as in Mucolipidosis (Type II, IIIA), or by the abnormalities in the lysosomal membrane protein LAMP2, due to the failure of communication with autophagosomes as in Danon disease (Platt et al., 2012). On the other hand, hyperresponsive lysosomes are also fatal to cells. Recent research of the regulated cell death (RCD) has revealed pathways for active lysosome-mediated cell death, termed lysosome-dependent cell death (LDCD) (Del Re et al., 2019; Mishra et al., 2019; Tang et al., 2019). LDCD is a type of RCD mediated by hydrolytic enzymes that are released into the cytosol after lysosomal membrane permeabilization (LMP). Lipid metabolites, such as sphingosine or ROS, increase the permeability of ions and hydrolases such as cathepsins to the cytosol, which initiate and amplify the cell death process. Indeed, cathepsins have been suggested to play a role in diabetic stress-induced cardiomyocyte death. Cathepsin D (CTSD) is known to activate the proapoptotic protein Bid, and is responsible for hyperglycemia-induced cardiomyocyte death (Kobayashi et al., 2020). Similarly, CTSB can induce NLRP3-mediated pyroptosis in the heart of type 1 diabetic mouse (Liu et al., 2022). Several other types of cell death, such as necrosis, have also been implicated in diabetic cardiomyopathy (Chen et al., 2020). However, it remains unknown if lysosomal dysfunction is involved in these types of cell death.

Accumulating evidence suggests that altered autophagy is often associated with lysosomal dysfunction (Pivtoraiko et al., 2009). The contribution of diminished lysosomal function to lysosomal storage disorders (Pivtoraiko et al., 2009), cardiac hypertrophy (Tang et al., 2009), and myocardial aging (Terman et al., 2008) has been well documented. Prior studies have also demonstrated lysosomal dysfunction in diabetic cardiomyopathy (Giacomelli et al., 1980; Skoza et al., 1980; Chua et al., 1983; Kutryk et al., 1987). For example, decreased lysosomal-associated membrane protein 2 (LAMP2) expression has been observed in the myocardium of type 2 diabetic mice, along with increased cardiomyocyte apoptosis (Xing et al., 2019). The cardiac injury was reversed with overexpression of LAMP2 in this diabetic mouse model. Saturated fatty acids (FAs), but not polyunsaturated FAs, decreased TFEB expression, induced aberrant lysosomal protein expressions such as Cathepsin B and LAMP2A, and increased proteotoxicity in cardiomyocytes (Trivedi et al., 2020).

In cardiomyocytes, the transcription factor EB (TFEB) plays a role in promoting autophagosome formation and lysosome biogenesis. There is evidence that TFEB is inactivated by phosphorylation in mice fed a high-fat, high-glucose diet, inhibiting its translocation to the nucleus, which leads to an inhibition of autophagosome formation, autophagosome-lysosome fusion, and lysosome biogenesis (Trivedi et al., 2020; Lu et al., 2021). Indeed, TFEB downregulation results in reduced levels of the coordinated lysosomal expression and regulation (CLEAR) network genes such as LAMP2A, Hsp90, and Hsc70 (Trivedi et al., 2016). Despite the association between lysosomal dysregulation and cardiac injury, it remains to be determined if lysosomal dysfunction can directly induce cardiac injury in diabetes.

## 5 LMP and ectopic release of lysosomal enzymes in diabetic cardiac injury

The autophagy-lysosomal degradation pathway is critical for maintaining cardiac homeostasis under both normal and pathologic conditions (Rothermel and Hill, 2007; Nishida et al., 2008). In fact, altered autophagy has been observed in both type 1 and type 2 diabetic mouse hearts (Mellor et al., 2011a; Mellor et al., 2011; Xie et al., 2011; Xu et al., 2013). However, it remains controversial how exactly autophagic activity is altered in the diabetic heart, as both decreased and increased cardiac autophagy have been reported in animal models of either type 1 (Xie et al., 2011; Xu et al., 2013; Yuan et al., 2016; Zhang et al., 2016) or type 2 diabetes (Mellor et al., 2011a; Kanamori et al., 2015; Trivedi et al., 2016). These discrepancies may be due to a multitude of differences in the animal models and research methods used, as discussed in a previous review (Kobayashi and Liang, 2015), including insulin deficiency or resistance, hyperglycemia and/or other changes associated with diabetes. Of note, not all studies have determined the autophagic flux in the heart, which may contribute to the variations among different reports. The lysosome is an independent organelle essential for cellular homeostasis in its own right. It is also required for autophagy to perform the degradative function. Thus, it is expected that lysosomal dysfunction will contribute to autophagic dysfunction. The studies that have reported lysosomal dysfunction and/or LMP in the heart of diabetic rodent animals are summarized in the Table 1. With a few exceptions (Nerurkar et al., 1988; Trivedi et al., 2016; Kobayashi et al., 2020), the number and activity of lysosomes tend to increase in type 1 (Chua et al., 1983; Kutryk et al., 1987; Kanamori et al., 2015; Guo et al., 2017; Liu et al., 2022) and decrease in type 2 (Giacomelli et al., 1980; Kuo et al., 1984; Kanamori et al., 2015; Xing et al., 2019) diabetes. Along with this observation, another question is whether lysosomal dysfunction in the heart of diabetic patients differs between males and females. Although most studies have used male animals (Chua et al., 1983; Kutryk et al., 1987; Nerurkar et al., 1988; Hua et al., 2013; Trivedi et al., 2016; Guo et al., 2017; Xing et al., 2019), studies using female animals (Giacomelli et al., 1980) and a mix of both sexes have shown similar results (Kobayashi et al., 2020; Liu et al., 2022), suggesting no sex difference in lysosomal dysfunction. However, an epidemiological survey has identified gender differences in the risk of cardiovascular disease and response to treatment in patients with diabetes and obesity (Peters et al., 2019). X-linked recessive lysosomal storage diseases such as Danon disease and Fabry disease manifest gender differences in the onset of cardiomyopathy that are more pronounced in males than females (Linhart et al., 2000; Meyer et al., 2014; Cenacchi et al., 2020). In addition, sex hormone receptor-responsive elements have been identified in human CTSD and TFEB genes (Shang et al., 2021). Thus, further investigation is warranted to elucidate the roles and the signaling mechanisms of sex hormones in the diabetic heart. Regarding lysosomal function in the diabetic heart, interventional experiments suggest that lysosomal proteases may be responsible for cardiac dysfunction in both type 1 and type 2 diabetes (Hua et al., 2013; Guo et al., 2017; Kobayashi et al., 2020; Liu et al., 2022). The changes in autophagy observed in the diabetic heart are likely to be dependent on fluctuations in lysosome activity. However, the exact relationship between lysosomal dysfunction and autophagy dysfunction remains to be explored in the diabetic heart.

**TABLE 1:** Studies demonstrating lysosomal dysfunction and/or LMP in the diabetic heart.

*In vivo* Diabetes	Model	Observations	References (PMID)
Type 1	Mouse (C57/BL6J), STZ-induced, sex unknown	Lysosomes are accumulated within cardiomyocytes	26042865 (Sesso et al., 2012)
	Rat (Sprague-Dawley), STZ-induced, male	Activity of cardiac lysosomal hydrolases are increased in the later stages of diabetes	2958002 (Pivtoraiko et al., 2009)
	Rat (Sprague-Dawley), alloxan-induced, male	Lysosomal Cathepsin D activity is upregulated in the diabetic heart	6869524 (Platt et al., 2012)
	Mouse (C57/BL6J), STZ-induced, mixed sex	Cathepsin D expression is upregulated in the diabetic heart	31862139 (Packer, 2020)
	Mouse (C57/BL6J), STZ-induced, male	Cathepsin K knockout protects against cardiac dysfunction in diabetes	28821796 (Skowyra et al., 2018)
	Mouse (C57/BL6J), STZ-induced, mixed sex	Cathepsin B is upregulated and deteriorates cardiac function in diabetic cardiomyopathy	36250207 (Paulson, 1997)
	Mouse (C57/BL6J), Ins2^Akita^ (Akita), male	Cathepsin B activity and LAMP2 expression are decreased, and TFEB inhibition is induced	27620487 (Sacco et al., 2003)
	Rat (Wistar, albino), STZ-induced, male	Cardiac Cathepsin D activity is initially reduced but later restored by insulin administration	3282952 (Shang et al., 2021)
Type 2	Mouse (C57BLKS/J), leptin receptor deficient (db/db), female and Mouse (C57BL/6), leptin deficient (ob/ob), female	Cardiac lysosomal hydrolase activity is reduced in association with the accumulation of residual bodies	6780237 (Radulovic et al., 2018)
	Mouse (C57BLKS/J), leptin receptor deficient (db/db), sex unknown	Lysosomes are rarely seen	26042865 (Sesso et al., 2012)
	Mouse (C57BLKS/J), leptin receptor deficient (db/db), sex unknown	Cardiac lysosomal protease (Cathepsin D) activity is decreased	6327362 (Skoza et al., 1980)
	Mouse (C57BL/6), STZ + HFD-induced, male	LAMP2 expression is decreased in the diabetic heart	31564413 (Reggiori et al., 2021)
	Mouse (C57BLKS/J), leptin receptor deficient (db/db), sex unknown	Cathepsin D expression is upregulated in the diabetic heart	31862139 (Packer, 2020)
	Mouse (C57BL/6), HFD-induced, male	Cathepsin K knockout mitigates HFD–induced cardiac hypertrophy and dysfunction	23069627 (Tan and Finkel, 2022)
	Mouse (C57/BL6J), High fat and high sucrose-induced obesity, male	Cathepsin B activity and LAMP2 expression are decreased, and TFEB inhibition is increased	27620487 (Sacco et al., 2003)

Abbreviations: STZ, streptozotocin; HFD, high-fat diet.

Severe lysosomal membrane damage eventually leads to the release of lysosomal proteases into the cytoplasmic compartment, triggering various types of cell death (Alu et al., 2020). In the heart of a type 1 diabetes mouse model, cathepsin B is released from lysosomes, which activates NLRP3, triggering pyroptosis (Liu et al., 2022). Lysosomes also plays a role in the regulation of other cell death pathways. For example, necroptosis is regulated by STUB1-mediated ubiquitylation and lysosome-dependent degradation of RIPK1 and RIPK3 (Seo et al., 2016a; Seo et al., 2016b). Ferroptosis, a form of cell death characterized by iron-dependent lipid peroxidation, can also be influenced by lysosomes which store the active catalyst Fe^2+^. Disruptions of lysosomal acidification and the release of iron caused by LMP can lead to the lipid peroxidation of lysosomal membranes and the release of CTSB, promoting cell death (Nagakannan et al., 2021). Cathepsins, a family of proteases within lysosomes, play a major role in cell signaling under pathological conditions (Tang et al., 2019). Previous studies have reported altered levels of lysosomal proteases in diabetic hearts, highlighting the importance of lysosomal regulation in diabetic heart disease. Some studies demonstrate increased levels of cathepsin (CTS) in response to diabetic stress. Increased circulating CTSD levels were found in patients with coronary heart disease (Naseem et al., 2005; Goncalves et al., 2016) and diabetes mellitus (Feron et al., 2009; Mellor et al., 2011b; Mellor et al., 2011; Goncalves et al., 2016; Liu et al., 2017; Hoes et al., 2020), which correlated with the severity of heart failure (Hoes et al., 2020). Another cross-sectional study in patients with type 2 diabetes and cardiovascular disease demonstrated increased serum CTSS levels. Patients from this study who also had acute coronary syndrome showed a marked increase in CTSS levels compared to patients with stable angina pectoris (Jing et al., 2021). Together, these data suggest that increased cathepsin levels are linked to cardiac injury in diabetic patients.

In animal models, CTSD activity has been found to be either reduced (Nerurkar et al., 1988) or increased (Kanamori et al., 2015; Kobayashi et al., 2020) in the hearts of streptozotocin-induced type 1 diabetic rats or mice. In db/db type 2 diabetic mice, CTSD was reported to be decreased in the heart at the initial stage (Kuo et al., 1984; Kanamori et al., 2015), but rebounding at a later stage when there was accelerated degradation of cardiac muscle (Kuo et al., 1984). The reduction of CTSD at the initial stage was speculated as an adaptive response (Kuo et al., 1984; Muller and Dhalla, 2012). Results from our former study also showed that CTSD was accumulated in the hearts of both type 1 and type 2 diabetic mouse models (Kobayashi et al., 2020). However, the functional role of CTSD in diabetic cardiomyopathy remains largely unknown, despite a previous study suggesting a protective role for CTSD in myocardial infarction (Wu et al., 2017). In this respect, we showed that high glucose (HG) altered lysosomal pH and induced LMP and lysosomal injury, which was associated with increased expression and abnormal distribution of CTSD, indicating leakage into the cytosol, in cardiomyocytes (Kobayashi et al., 2020). Importantly, CTSD knocking down with siRNA or inhibiting CTSD activity by pepstatin A reduced HG-induced cardiomyocyte death, while CTSD overexpression exacerbated HG toxicity (Kobayashi et al., 2020). These results support the possibility that the increased LMP and the ensuing CTSD leakage and aberrant accumulation of CTSD may cause cardiomyocyte injury, contributing to heart failure in diabetes. If this is true, enhancing lysosomal quality control and minimizing the ectopic effects of CTSD would be expected to protect the diabetic heart. However, the only direct experimental evidence for a functional role of LMP and cathepsins in the process is obtained in isolated cardiac myocytes cultured under hyperglycemic conditions (Kobayashi et al., 2020), which is insufficient evidence to support the contention that LMP occurs in the diabetic hearts and is of functional consequence. Nevertheless, a detrimental role of CTSK in the diabetic heart has been demonstrated in streptozotocin-induced type 1 diabetes using CTSK knockout mice (Guo et al., 2017). Since CTSK knockout also reduced blood glucose levels, it is unclear whether the improvement in cardiac function is due to a direct effect of CTSK itself on cardiomyocytes or is related to the reduction in blood glucose levels (Guo et al., 2017). Together, these studies suggest that certain cathepsins such as CTSD and CTSK may contribute to diabetic cardiac injury, although further studies are needed to show the occurrence of LMP in the diabetic heart and to elucidate the underlying molecular mechanisms.

## 6 Summary and future perspectives

The lysosome is an essential organelle by which autophagy turns over proteins and organelles to maintain cellular homeostasis. Lysosomal dysfunction and altered activities of lysosomal cathepsins have been observed in the diabetic heart. However, it is currently unknown whether lysosomal membrane permeabilization (LMP) and the ensuing cathepsins release are responsible for diabetic cardiac injury. An emerging hypothesis postulates that the increased LMP and the ensuing cathepsins leakage and aberrant accumulation are an important mechanism that mediates diabetic cardiomyopathy and heart failure; thus, enhancing lysosomal quality control and minimizing the ectopic effects of cathepsins would be expected to protect the diabetic heart. To test this hypothesis, one needs to address a number of different questions, including: What causes LMP in diabetic myocardium? What is the extent of lysosomal damage caused by diabetic stress? Are there biomarkers to assess such damage? How do cardiomyocytes repair lysosomal membrane damage caused by diabetes? Which and how cathepsins affect cardiomyocyte death and survival? Further investigation is warranted to determine the exact functional roles of these cathepsins in the diabetic heart by using pharmacological agents and/or genetic animal models with increased or decreased activities of each of the cathepsins. Moreover, future studies should also determine whether ectopic localization of these potential targets occurs in the diabetic heart and how the LMP repairing mechanisms and lysophagy respond to these changes. Addressing all the above issues will help elucidate the mechanisms underlying diabetic cardiomyopathy and suggest novel strategies for restoring lysosomal function and reducing diabetic cardiac injury.
